# Visualising Viruses

**DOI:** 10.1099/jgv.0.001730

**Published:** 2022-01-27

**Authors:** Annabel Slater, Naina Nair, Rachael Suétt, Rian Mac Donnchadha, Connor Bamford, Seema Jasim, Daniel Livingstone, Edward Hutchinson

**Affiliations:** ^1^​ School of Life Sciences, University of Glasgow, Glasgow, UK; ^2^​ School of Simulation and Visualisation, The Glasgow School of Art, Glasgow, UK; ^3^​ School of Engineering, University of Glasgow, Glasgow, UK; ^4^​ MRC-University of Glasgow Centre for Virus Research, Glasgow, UK; ^†^​ Present address: Wellcome-Wolfson Institute for Experimental Medicine, Queen’s University Belfast, Belfast, Ireland

**Keywords:** SARS-CoV-2, influenza virus, visualisation

## Abstract

Viruses pose a challenge to our imaginations. They exert a highly visible influence on the world in which we live, but operate at scales we cannot directly perceive and without a clear separation between their own biology and that of their hosts. Communication about viruses is therefore typically grounded in mental images of virus particles. Virus particles, as the infectious stage of the viral replication cycle, can be used to explain many directly observable properties of transmission, infection and immunity. In addition, their often striking beauty can stimulate further interest in virology. The structures of some virus particles have been determined experimentally in great detail, but for many important viruses a detailed description of the virus particle is lacking. This can be because they are challenging to describe with a single experimental method, or simply because of a lack of data. In these cases, methods from medical illustration can be applied to produce detailed visualisations of virus particles which integrate information from multiple sources. Here, we demonstrate how this approach was used to visualise the highly variable virus particles of influenza A viruses and, in the early months of the COVID-19 pandemic, the virus particles of the then newly characterised and poorly described SARS-CoV-2. We show how constructing integrative illustrations of virus particles can challenge our thinking about the biology of viruses, as well as providing tools for science communication, and we provide a set of science communication resources to help visualise two viruses whose effects are extremely apparent to all of us.

## Introduction

Viruses compel our attention and frustrate our imagination. The impact of pathogenic viruses on everyday life is all too easy to observe, but viruses themselves are not a part of the visible world. This is partly because, like all microorganisms, they operate on a scale too small for the unaided eye to see, but an even greater challenge in forming a clear image of viruses is the intimacy with which they parasitise their hosts. Unlike parasitic cellular microorganisms such as bacteria or eukaryotic microbes, viruses are not discrete bodies that draw nutrition from their hosts [1]. Instead, the replication cycle of a virus involves large populations of closely-related nucleic acids propagating through, diffusing among and interacting directly with the teeming molecular complexity found within and between host cells. Rather than picturing this mingling of molecules through multiple microscopic compartments, we typically use high-level abstractions such as the flow of genetic information, the activation of signalling pathways and the accumulation of infectious particles. These are useful, but they do not lend themselves to direct visual representation.

It is often helpful, both for the wider public and for virologists themselves, if the term ‘virus’ can be grounded in a more concrete mental image. To do this, we conventionally turn to the one part of the virus replication cycle that has a discrete and distinctive physical form, and which clearly separates viruses from other symbiotic nucleic acids such as plasmids or transposons [2]. When they think of ‘viruses,’ most people will imagine the virus particles (or ‘virions’) that carry viral genomes from one cell the next. Because of this, clear visual representations of virus particles are important tools for communicating about viruses. However, for many viruses, including many of medical importance, accurate models of their virus particles are difficult to assemble. This article discusses how methods from medical visualisation can tackle the challenge of creating a concrete visual representation of an invisible virus, even when a complete experimental description of the virus particle is lacking. To do so we will look at examples from two clinically important viruses – the influenza viruses and severe acute respiratory syndrome coronavirus 2 (SARS-CoV-2).

## Why we visualise virus particles

Virus particles are only one step in the virus replication cycle, and for many viruses they are absent during most stages of viral replication. Nonetheless, the image of a virus particle is helpful when thinking about viruses. As noted above, the production of virus particles is one of the characteristic features of viruses, distinguishing them from other symbiotic nucleic acids [2]. The size of virus particles, on the ‘mesoscale’ intermediate between the sizes of macromolecules and the sizes of subcellular structures, draws attention to the length scales in which individual viruses and their components operate [3]. The infectious nature of virus particles means that their properties are key to understanding infectivity, transmissibility, tropism and neutralisation by antibodies, which in turn gives them a central role in effective public communication about controlling viruses through both non-pharmaceutical interventions and vaccination. Finally, the mental image of a virus particle is compelling. Their beauty and elegance locate the invisible threat of viruses as a part of the natural world, encourage us to think of viruses as being just as comprehensible as the biology that we can observe directly, and draw people to study viruses in more detail. For all these reasons, it is unsurprising that virus particles have become emblematic of virology as a whole. Images of virus particles are used widely in everything from media reports on viral outbreaks to the brand identity of specialist society journals. Being able to imagine a virus is the entry point for discussions of virology, but building a good visual model of a virus particle is often extremely challenging.

## Challenges in visualising virus particles

The first challenge in visualising a virus particle is that the question ‘what does a virus particle look like?’ does not actually have a direct answer. Virus particles are typically smaller than the wavelength of visible light, undergo constant thermal motion at speeds our brains are not well-equipped to deal with, and are small enough that their shape is defined by the electrostatic properties of individual molecules. There are, however, well-established conventions for simplifying these issues. Most people can interpret a visual model that answers the alternative question ‘what would a virus particle look like if it was enormously bigger in size, moved very slowly if at all, and was made of components with clearly-defined edges?’ To address this simpler question we still have the challenge of defining the shapes of a virus particle’s constituent macromolecules and how they relate to each other. There are currently two ways to do this.

The most direct approach to visualise a virus particle is from experimental measurements. One of the striking features of many virus particles, particularly those of non-enveloped viruses, is their strict regularity. This has been exploited by structural biologists, who can use the large numbers of identical virus particles present on electron microscopy grids or, implicitly, in diffracting crystals to generate high-resolution average structures. Such structures are strikingly beautiful and often contain enough structural information to build testable models of viruses as molecular machines [4, 5].

However, direct experimental measurement is often not sufficient. Many viruses, particularly those bound by a lipid membrane, are pleiomorphic, or continuously variable in form. A population of such particles will show considerable variation in their dimensions and proportions, and no two virus particles will be entirely identical – the ideal, average, virus particle may or may not even exist. In such cases, experimentally averaged structures provide limited detail. Instead, a model of the virus particle has to be obtained by integrating data from multiple sources. This is typically done by fitting the structures of individual molecules, determined experimentally or predicted by modelling, into low-resolution electron micrographs or tomograms of individual virus particles.

## Integrative models of virus particle structure

The synthesis of diverse data sources to understand complex biological structures that are inaccessible by any one method is the subject of the emerging field of integrative structural biology [6, 7]. Integrative methods are beginning to provide striking insights into the vast and variable macromolecular assemblies that mediate biological processes such as nuclear transport [8], membrane trafficking [9] and secretion [10]. Virus particles are appealing targets for such approaches, and in principle integrative models of virus particles should be both effective visual aids and sources of testable structural models [11–14].

However, the application of rigorous integrative methods to variable virus particles is not trivial. Integrative modelling of virus particles requires rich datasets: to identify and quantify the proteins encoded by both the virus and its host that are packaged into virus particles; to precisely define their individual and higher-order structures; and to map their interactions and the organisation of the virus particle as a whole. Often this information is not sufficiently complete for a rigorous mesoscale model to be produced. The vast majority of viruses are not yet studied in sufficient detail [15]. Some viruses, such as hepatitis C virus, produce virus particles that are particularly hard to purify and study [16]. And during a crisis, such as the current SARS-CoV-2 pandemic, there is a demand to visualise a virus when it is still in the early stages of being studied.

In such cases, the ‘visualisation gap’ between individual molecular structures and overall virion morphology can be tackled using artistic approaches informed by scientific data. Scientific illustration has long been effectively applied to virology, both to communicate established models of viruses effectively to a wider audience and to challenge scientific thinking about the way viruses function [17]. We recently formed collaborations between virologists and biomedical illustrators to build visual models of the virus particles of two intensively-studied viruses, influenza A viruses and SARS-CoV-2. We found that building even a loosely-constrained model of a virus particle can both challenge our assumptions about virology and be a flexible and stimulating resource to support communication about viruses.

## Modelling influenza virus particles

Influenza viruses are major human and veterinary pathogens [18]. In humans, influenza A and B viruses cause a seasonal respiratory infection which in typical years causes hundreds of thousands of deaths, sickens many more and causes enormous economic losses [19]. In addition, influenza A viruses (IAV) have an unusual facility for crossing between host species and cause regular pandemics, including the ‘Great Influenza’ of 1918 which is among the worst infectious disease outbreaks on record. Human influenza has long been established as a viral illness, with the infectious agent identified as a virus in 1933 [20, 21]. Due to their clinical and veterinary importance, and because they grow well in laboratory systems, influenza viruses have been intensively studied for decades. However, there have been relatively few attempts to integrate the findings of this work into a detailed and accurate model of an influenza virus particle [14, 17, 22–24].

One challenge is that influenza viruses, as studied in the laboratory, are rather more regular than those that occur in natural infections. Laboratory-adapted strains of influenza virus typically form spherical or bacilliform virions of around 120 nm diameter, but low-passage clinical isolates can form additional, filamentous virus particles that are slightly narrower and can extend for microns in length, reaching tens to hundreds of times the length of the spherical particles [25, 26]. These filamentous influenza virions are poorly-studied and are only rarely represented in images of influenza virus particles.

**Fig. 1.**
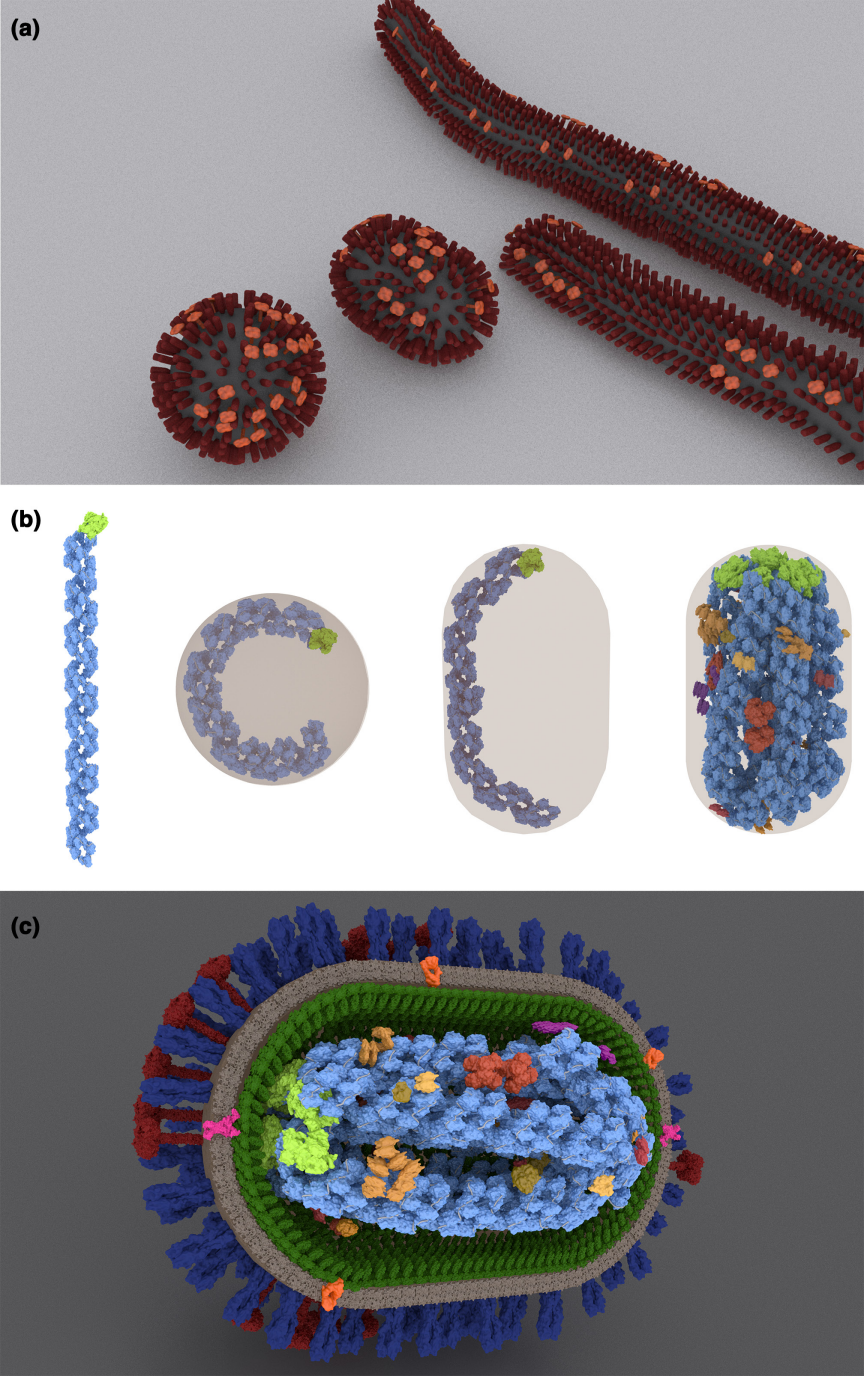
Modelling influenza A virus particles. (**a**) Variable morphologies of influenza A virus (IAV) particles, based on cryoelectron tomography of the strain A/Udorn/307/1972(H3N2). (**b**) A visual argument that the ribonucleoproteins (RNPs) encapsidating the longest IAV genome segments could only fit within a spherical virus particle of 120 nm outer diameter (of 80 nm internal diameter, shown in brown) if severely contorted, but that a bacilliform virus particle of equivalent surface area has the capacity to enclose the RNPs with minimal contortion, allowing them to form a parallel array. (**c**) A detailed model of a bacilliform IAV virus particle showing proteins encoded by both the virus and its host. Host proteins and membrane are in brown, reds and oranges; viral proteins are polymerase trimer (light green) and NP (light blue) in the RNPs; and HA (blue), NA (red), M2 (pink), M1 (dark green), NEP and NS1 (both purple).

To address this, we built models of intact influenza virus particles using Autodesk 3ds Max (Autodesk; Fig. 1a). These were constructed at low resolution, as capsules decorated with simple geometric models of the surface proteins haemagglutinin (HA) and neuraminidase (NA), spaced in a similar way to published electron tomograms [27–30]. Virus particle shapes were based on published cryoelectron tomograms of the IAV strain A/Udorn/307/1972(H3N2) [31], with dimensions representative of the three classes into which the continually variable influenza virions are divided: spherical, bacilliform and filamentous (Table S1, available in the online version of this article) [25, 31].

We next set out to construct a more detailed model of an individual influenza virus particle, working in Autodesk 3ds Max using data collected from non-filamentous laboratory strains. The protein composition of virus particles was based on a mass spectrometry proteomics study we had previously carried out using influenza A/WSN/33(H1N1) particles grown in Madin Darby Bovine Kidney cells and purified on chicken erythrocytes [32]. As this method is known to systematically underestimate the abundance of glycoproteins [33] the absolute numbers of HA and NA were increased (in a constant ratio) to match the surface density reported in cryoelectron tomograms [27] (Table S2, available in the online version of this article). Protein structures (Table S2) were mainly based on experimentally-determined structures from the Protein Data Bank, with structural prediction by QUARK [34, 35] used for viral proteins whose structures were unknown or lacked domains. As in a previous study [24], the stem of NA was modelled using an unrelated alpha helix. The cross-section of the membrane was based on a molecular dynamics simulation of a lipid bilayer [36], and viral RNA was modelled as a simple tube. Ribonucleoprotein complexes (RNPs) were assembled based on cryo-electron tomograms [37, 38], assuming that each NP monomer bound approximately 24 nt of RNA [39]. This gave RNP lengths that were a good match to experimental measurements, ranging from 50 to 150 nm depending on the length of the genome segment [37, 38, 40]. Host proteins were included if mass spectrometry suggested that they were present at more than one copy per virion (assuming eight polymerase complexes per virion) and if a protein structure of the protein or a homolog was available in the Protein Data Bank [32]. Protein structures were imported into Autodesk 3ds Max using the Molecular Maya 2016 plugin.

We had initially intended to create a detailed model of a spherical virus particle of 120 nm external diameter, a commonly reported form of laboratory-adapted virion and the shape most often used in illustrations of influenza virus particles [23]. However, when manually modelling the particle we realised that accommodating RNPs inside this particle, which would have an internal diameter of approximately 80 nm, would introduce severe distortions into the longer RNPs, to an extent that appeared incompatible with maintaining the ‘7+1’ parallel array of RNPs observed in budding virions (that is to say, seven RNPs surrounding one central RNP; Fig. 1b) [27, 41–45]. This initially surprised us, but a review of the literature suggested that most influenza virus particles in which 7+1 arrays were described were either in the process of budding, were elongated (bacilliform or filamentous) or were of uncertain morphology (as when imaged in thin sections). Indeed, a study that used cryoelectron tomography to segment a spherical virus particle, for the structurally similar influenza B virus, found a contorted arrangement of RNPs rather than a parallel array of RNPs [46]. We noted that, because bacilliform virions are typically somewhat narrower than spherical virions, the surface area of both morphologies will in fact be similar (Table S1, available in the online version of this article; calculated from data in [25]), suggesting that one could deform into the other. On this basis, we propose that models showing 7+1 arrays of RNPs within spherical influenza virus particles are typically misleading, as only a small proportion of spherical influenza virus particles are large enough to avoid severely contorting the RNPs until they are no longer in a parallel array. We also speculate that spherical particles may be produced by the collapse of ‘naturally’ bacilliform particles into a lower-energy spherical shape following budding.

With this information, we were able to construct a detailed three-dimensional model of an influenza virus particle, incorporating the structures of both virus and host proteins (Fig. 1c). Constructing the model challenged some of our initial assumptions about influenza virus particle structure, but also indicated that the high levels of host proteins observed in influenza virus particles could feasibly be accommodated in a particle of this size [32].

## Modelling SARS-CoV-2 particles

**Fig. 2.**
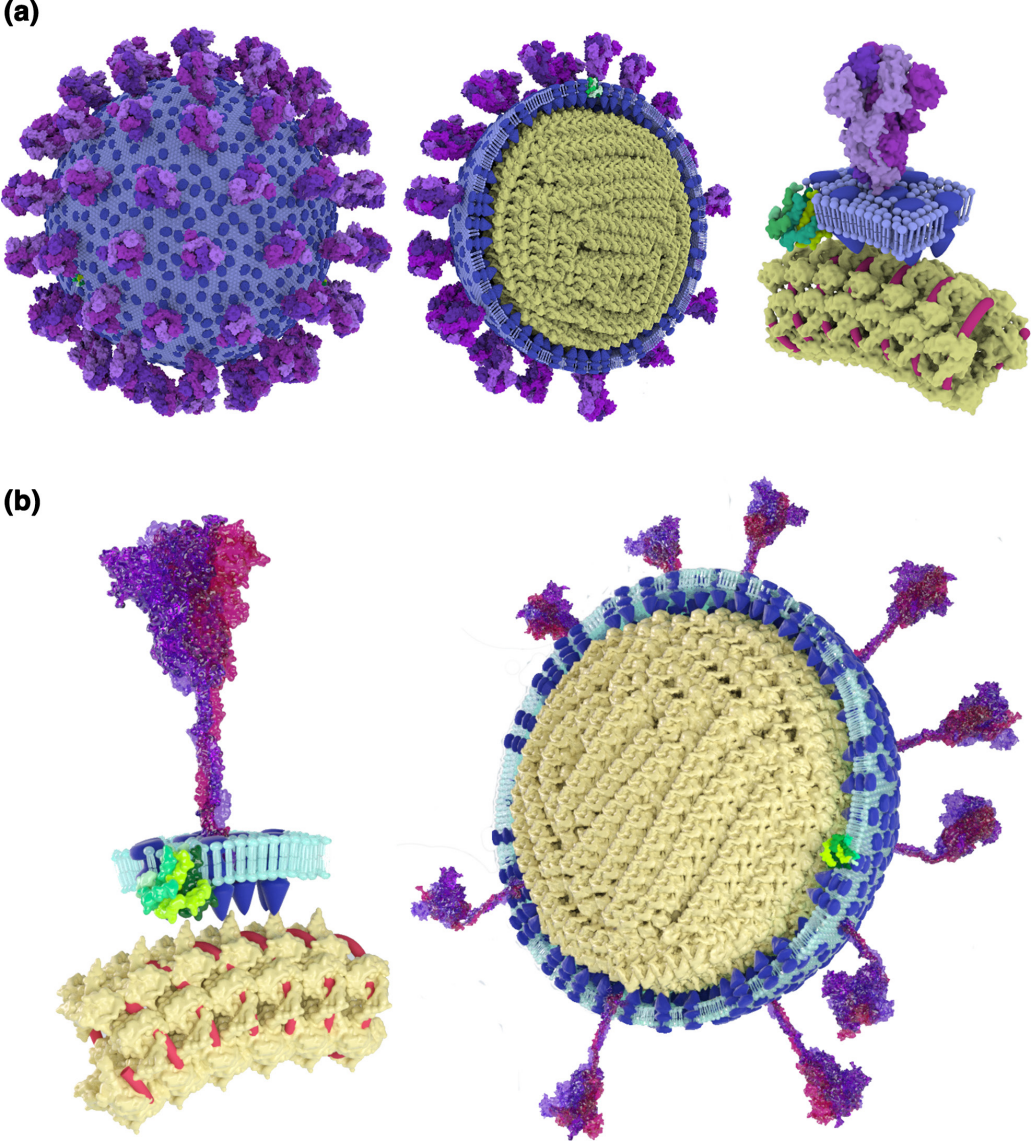
Modelling SARS-CoV-2 virus particles. (**a**) An initial model of a SARS-CoV-2 virion, produced in the early months of the COVID pandemic and partly based on studies of related coronaviruses. S proteins are purple, M proteins blue, E proteins green, N proteins yellow, and genomic RNA red. (**b**) A revised model incorporating information from experimental studies of SARS-CoV-2.

In response to the need for effective science communication in the early days of the SARS-CoV-2 pandemic, we sought to apply a similar literature-informed illustrative approach to constructing a model of the SARS-CoV-2 virus particle. In order to illustrate a virus particle whose basic features were, in March 2020, still being worked out, we drew on literature describing the dimensions, stoichiometry and structural organisation of virus particles of the closely-related SARS-CoV-1 (SARS-CoV) [47–50] as well as on studies of the more distantly-related betacoronavirus murine hepatitis virus [51] (Table S3, available in the online version of this article). We based the E protein structure on NMR data from its orthologue in SARS-CoV-1 [52], illustrated the M protein in Autodesk 3ds Max using data from an electron microscopy study of SARS-CoV-1 [53], and modelled the N protein and overall RNP organisation using experimental studies of SARS-CoV-1 [48, 49], infectious bronchitis virus [54], murine hepatitis virus [51] and a SARS-CoV-2 protein structure predicted by I-TASSER [55]. Lipids were modelled as simplified geometrical forms. These data, along with the publication of a structure for the SARS-CoV-2 spike protein [56] allowed us to construct an initial model of a spherical virion of 120 nm external diameter (88 nm outer membrane diameter). This model (Fig. 2a), whose external features resemble the widely-used visualisation of the SARS-CoV-2 particle developed by Alissa Eckert and Dan Higgins of the US Centres for Disease Control [57], was one of the first to attempt to represent the internal features of the SARS-CoV-2 virus particle as well as its surface structures. In response to the rapidly growing body of literature on SARS-CoV-2 we later updated the model, drawing on a model that more accurately represented the flexible stem of the spike protein [58], and on cryoelectron tomography studies [59, 60] which showed that spike proteins were present at a lower density on the surface of SARS-CoV-2 than we had predicted from studies of murine hepatitis virus and SARS-CoV-1 [49, 51], and that the spike proteins could bend both with respect to the membrane and along their stems (Fig. 2b).

As more information is acquired about SARS-CoV-2, there is a growing potential for developing integrative models of its virus particles, as their features can be placed under detailed experimental constraints [12, 13, 61]. However, in the early stages of the pandemic a fully data-driven approach was not possible. Instead, illustrative techniques allowed us use comparative virology to fill in the gaps in our mental image of the virus particle.

## Seeing improvements

The intense interest in SARS-CoV-2 means that our model is now only one of a large number of visualisations showing the virus particle in different levels of detail [13, 57, 62–64]. There are also a smaller but growing number of visualisations of influenza virus particles [14, 17, 22–24]. It is to be expected that increasingly detailed and accurate models will be developed as more studies of both viruses are carried out. We see potential for increasing the detail and accuracy of visualisations of virus particles in five main areas:

- An increased level of detail. For example, by including glycans, which can block antibody binding as well as regulating receptor binding interactions [58].
- The refinement of specific details. For example, the helical structure of the influenza RNP was initially solved with ambiguous handedness [37, 38], and is shown in our models as left-handed, but recently-published work suggests that the RNP helix may be right-handed [65]. As discussed above in the context of influenza viruses, details of the overall arrangement of the genome within the particles of both SARS-CoV-2 and influenza viruses are still emerging.
- Visualising diversity. Visualisations of virus particles tend to focus on a single idealised form, and the heterogeneity of virus particles produced by both influenza and SARS-CoV-2 virus particles is rarely shown.
- Visualising dynamics. Virus particles are typically visualised as static structures, but they undergo constant thermodynamic motion and specific conformational changes that are relevant to their function.
- Visualising context. While most visualisations of virus particles consider them in isolation, in reality they are part of, and interact with, a complex and crowded molecular environment. In the case of SARS-CoV-2 and influenza virus particles, even when airborne, this environment is typically respiratory mucus [62, 66].

However, it is important to recognise that the best level of detail and accuracy in any visualisation is not necessarily the greatest level possible. Good visualisations are a form of communication, and therefore include design choices that reflect the needs and expectations of their intended audience. When putting visualisations of virus particles to use, the needs of different audiences can vary considerably.

## Putting visualisations to use

As discussed above, the process of constructing integrative illustrations of the virus particles of influenza virus and SARS-CoV-2 was directly helpful to us as virologists. Doing so required us to synthesise ideas, challenged our preconceptions, and encouraged us to extend our own thinking about the molecular biology of these viruses. The primary purpose of this work, however, was to support science communication with others. In the time since the models have been constructed this has taken place in three distinct ways (Table 1).

**Table 1.** Uses of visualisations of virus particles

Use	Examples
Decoration	Professional websites: MRC PPU and CVR Coronavirus Toolkit https://mrcppu-covid.bio/ MRC-University of Glasgow Centre for Virus Research https://www.gla.ac.uk/researchinstitutes/iii/cvr/ CoV-GLUE http://cov-glue.cvr.gla.ac.uk/ Reports: UNEP report: Preventing the next pandemic [67]
Communication	Articles: SARS-CoV-2 https://coronavirusexplained.ukri.org/en/article/cad0010/ Influenza viruses [18] Educational Resources Colouring sheets [68]; *see also supplementary information* *Visible Viruses* augmented reality app https://cvrblog.myportfolio.com/visible-viruses-ar-app Online 3D models of influenza A virus https://sketchfab.com/3d-models/a-variety-of-influenza-virus-particles-9853fc3934c6435a9d46857e61428934 https://sketchfab.com/3d-models/influenza-virus-b3ef4264dbcf4d59a75b3c551484fc96 Online 3D models of SARS-CoV-2 https://sketchfab.com/3d-models/sars-cov-2-half-virion-updated-141250900aad4494a542d4e258b35a00 https://sketchfab.com/3d-models/sars-cov-2-virion-updated-f3235b3b7bc949958ce41b83825ae384
Imagination	3D print designs of SARS-CoV-2 https://www.thingiverse.com/thing:4687896 https://www.thingiverse.com/thing:4973130 https://www.thingiverse.com/thing:4973133 3D print design of influenza A virus, available at https://researchdata.gla.ac.uk/1220

**Fig. 3.**
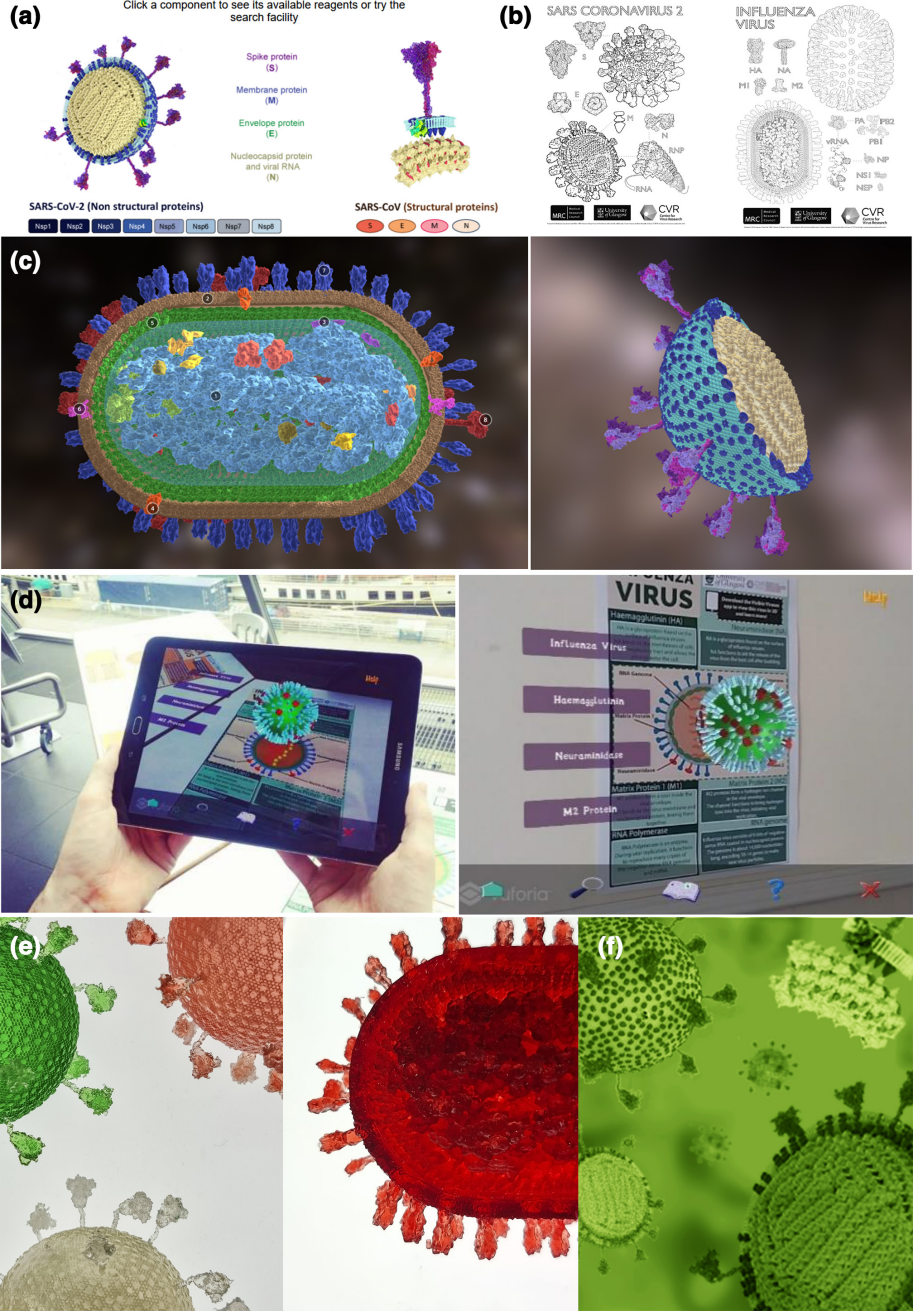
Using visualisations of virus particles in science communication. A selection of uses of the virus particle models described in this paper. (**a**) As branding for a virology reagent website (the *MRC PPU and CVR Coronavirus Toolkit*; image used with permission), (**b**) in educational colouring sheets, (**c**) as interactive 3D models on *SketchFab* (**d**) in the augmented reality app *Visible Viruses*, (**e**) as 3D prints and (**f**) as branding for the *Journal of General Virology*.

The most straightforward use of the models, as for any visualisation, was decorative. As well as being visually appealing, such models provide an immediate indication that a resource relates to viruses. The style in which the virus particle is represented adds nuance to this – while simplified designs can be used to indicate widely accessible content, very detailed visualisations of virus particles convey credibility and can be used to highlight that a resource is aimed at engaged or expert audiences. Our visualisations were used in this way in the websites of virology departments, virology resources and PhD programmes, as well as being included in literature discussing the pandemic (Fig. 3a).

Visualisations of virus particles can also be used more deliberately as resources for science communication. The level of detail in these structures means that they can be used to explain the relationship between the underlying molecular biology of these viruses and their impact on our lives – for example, in discussing which parts of the viruses are the targets of neutralising antibodies, or to show why these viruses can be inactivated by alcohol or detergents. The models were used in this way in articles explaining the virus to the general public and in colouring sheets intended for home study and science festivals (Fig. 3b). They were also made available for self-directed exploration in *Sketchfab*, an online resource which allows three-dimensional structures to be explored in web browsers and in virtual reality, and which provides the potential for them to be embedded in virtual reality teaching resources (Fig. 3c). We also incorporated the models into an educational app that allows virus structures to be explored on mobile devices using augmented reality (Fig. 3d).

Finally, visualisations of virus particles can stimulate creative projects in response to the pandemic. Discussion of the models on social media led to their use as the basis for a set of 3D printed virus particles (Fig. 3e), and the design team of the *Journal of General Virology* has now used them as the basis for an update to this journal’s branding (Fig. 3f).

We hope that these models will continue to provoke other people to reimagine and respond to the invisible viruses that have such an impact on all of us. The models themselves and a set of materials produced from them can be accessed through the links and supplementary information provided with this article. Visualising a virus particle is only one step in trying to understand how viruses are woven through our world. But it is often the step that opens up other aspects of the virus to our imagination and, by using illustrative approaches to look ahead of our experimental descriptions, we can think more clearly about the very visible impact that viruses have on all of us.

## Additional resources

Additional resources accompanying this article (high resolution image files, animations, educational colouring sheets and model files for the virus structures) can be downloaded from the University of Glasgow’s Enlighten Data Repository at https://researchdata.gla.ac.uk/1220.

## Supplementary Data

Supplementary material 1
