# Supplementary material for: Visualising Viruses
Source: J Gen Virol. 2022 Jan 27;103(1):001730. doi: 10.1099/jgv.0.001730 (PMC8895616; doi:10.1099/jgv.0.001730)
Supplement: Supplementary material 1 [file jgv-103-1730-s001.pdf]

Visualising Viruses

Supplementary Tables

Annabel Slater[1], Naina Nair[2], Rachael Suétt[2], Rian Mac Donnchadha[3], Connor Bamford[4,5], Seema Jasim[4], Daniel Livingstone[2] and Edward Hutchinson[4]*

[1]School of Life Sciences, University of Glasgow

[2]School of Simulation and Visualisation, The Glasgow School of Art

[3]School of Engineering, University of Glasgow

[4]MRC-University of Glasgow Centre for Virus Research

[5]Current address: Wellcome-Wolfson Institute for Experimental Medicine, Queen's University Belfast

*Edward.Hutchinson@glasgow.ac.uk

Supplementary Table 1: Dimensions of influenza A virus particles

Morphology	Typical dimensions, including surface glycoproteins (nm)*	Typical outer dimensions of membrane (nm)	Typical outer membrane surface area (nm2)**	Typical inner dimensions (within the membrane and layer of bound matrix protein***) (nm)
Spherical	120×120	100 × 100	31000	80 × 80
Bacilliform	140 × 95	120 × 75	28000	100 × 55
Filament	80 × 250 – 30,000+	60 × 230 – 30,000+	variable	40 × 210 – 30,000+

* After Dadonaite *et al.* (2016) *JGV* 97(8) 1755-1764

** Surface area of a sphere of radius a is $4\pi a^2$; surface area of a bacillus (a cylinder capped by hemispheres) of long axis $2a$ and short axis $2b$ is $4\pi ab$.

***A lipid bilayer bound to a layer of M1 matrix protein is taken to have a combined thickness of c.10 nm, as e.g. Harris *et al*. (2006) *PNAS* 103 19123–19127.

Supplementary Table 2: Composition of influenza A virus particles

Origin	ID	Name	Short	Number (protein)	PDB	Presumed localisation
**Viral**	M1	Matrix Protein	M1	2106	1EA3 + modelled C-terminus	helical polymer bound to inner face of membrane
	NP	Nucleoprotein	NP	535 (in 8 RNPs of varying length)	4BBL	RNP: double helix with RNA, capped by PB2, PB1 and PA (the ribonucleoprotein; RNP)
	HA	Haemagglutinin	HA	296 (as 99 trimers)	1RU7	protruding from membrane
	NA	Neuraminidase	NA	44 (as 12 tetramers)	3BEQ head + 1GCL stalk	protruding from membrane
	M2	M2	M2	9	2L0J plus modelled N- and C-termini	transmembrane
	NS1	Non-structural Protein 1	NS1	1	4OPH	intracellular
	PA	Polymerase Acidic	PA	8 (in 8 RNPs)	4WSB (PB2, PB1 and PA)	part of the RNP
	PB1	Polymerase Basic 1	PB1	8 (in 8 RNPs)	4WSB (PB2, PB1 and PA)	part of the RNP
	PB2	Polymerase Basic 2	PB2	8 (in 8 RNPs)	4WSB (PB2, PB1 and PA)	part of the RNP
	NEP	Nuclear Export Protein	NEP	5	Modelled	intracellular
**Host**	P21926	CD9 antigen	CD9	12	5TCX (CD81)	transmembrane
	P07355	Annexin A2	ANXA2	6	1W7B	intracellular
	P60709	Actin, cytoplasmic 1	ACTB	5	1J6Z	intracellular
	P04083	Annexin A1	ANXA1	4	1HM6	intracellular
	P62988	Ubiquitin	UBB	3	1UBI	intracellular
	P69905	Haemoglobin subunit alpha	HBA1	3 (as 1 tetramer)	5NI1	intracellular
	P07737	Profilin-1	PFN1	2	2BTF	intracellular
	P04406	Glyceraldehyde-3-phosphate dehydrogenase	GAPDH	2	4WNC	intracellular
	P02100	Hemoglobin subunit epsilon	HBE1	2 (as 1 tetramer)	as HBA1	intracellular
	P14618	Pyruvate kinase isozymes M1/M2	PKM	2 (as 1 dimer)	4XFJ	intracellular
	P63104	14-3-3 protein zeta/delta	YWHAZ	1	4HKC	intracellular
	P37802	Transgelin-2	TAGLN2	1	1WYM	intracellular
	P63000	Ras-related C3 botulinum toxin substrate 1	RAC1	1	3TH5	intracellular
	Q6LES2	Annexin A4	ANXA4	1	2ZOC	intracellular
	P23528	Cofilin-1	CFL1	1	1Q8G	intracellular
	P12429	Annexin A3	ANXA3	1	1W7B	intracellular
	P13928	Annexin A8	ANXA8	1	1W7B	intracellular
	P11142	Heat shock cognate 71 kDa protein	HSPA8	1	4H5T	intracellular
	P60953	Cell division control protein 42 homolog	CDC42	1	2KB0	intracellular
	Q9BUF5	Tubulin beta-6 chain	TUBB6	1	4HNA	intracellular
	Q13509	Tubulin beta-3 chain	TUBB3	1	4HNA	intracellular
	P62937	Peptidyl-prolyl cis-trans isomerase A	PPIA	1	3K0M	intracellular

	P61586	Transforming protein RhoA	RHOA	1	3T06	intracellular
**Membrane**	DPPC128			N/A	DPPC128 from: http://people.ucalgary.ca/~tieleman/download.html	membrane

Supplementary Table 3: Composition of SARS-CoV-2 virus particles

**Protein**	**Estimated copy number**	**Protein model**
**Spike peplomer (trimer)**	70 - 90	Initially 6VXX; later the model of Caslino et al. (2020)
**M (dimer)**	1100	Modelled at low resolution, after Neuman et al. (2006)
**N**	c.1500	QHD43423, modelled using D-I-TASSER/C-I-TASSER at https://zhanggroup.org/COVID-19/
**E**	20	5X29

Literature consulted:

Beniac DR, Andonov A, Grudeski E, Booth TF. Architecture of the SARS coronavirus prefusion spike. Nat Struct Mol Biol 2006;13:751.

Chang CK, Hou MH, Chang CF, Hsiao CD, Huang TH. The SARS coronavirus nucleocapsid protein – Forms and functions. Antiviral Res 2014;103:39–50.

Neuman BW, Adair BD, Yoshioka C, Quispe JD, Orca G, et al. Supramolecular Architecture of Severe Acute Respiratory Syndrome Coronavirus Revealed by Electron Cryomicroscopy. J Virol 2006;80:7918–7928.

Masters PS, Perlman S. Coronaviridae. In: Fields BN, Knipe DM, Howley PM (editors). Fields Virology. Philadelphia: Lippincott Williams & Wilkins; 2013. pp. 825–858.

Bárcena M, Oostergetel GT, Bartelink W, Faas FGAA, Verkleij A, et al. Cryo-electron tomography of mouse hepatitis virus: Insights into the structure of the coronavirion. Proc Natl Acad Sci U S A 2009;106:582–587.

Casalino, L., Gaieb, Z., Goldsmith, J., Hjorth, C., Dommer, A., & Harbison, A. et al. (2020). Beyond Shielding: The Roles of Glycans in the SARS-CoV-2 Spike Protein. ACS Central Science, 6(10), 1722-1734. doi: 10.1021/acscentsci.0c01056
